# Dietary Patterns of Children from the Amazon Region of Ecuador: A Descriptive, Qualitative Investigation

**DOI:** 10.3390/children10091568

**Published:** 2023-09-18

**Authors:** Kristin N. Murphy, Lisa K. Boyce, Eduardo Ortiz, Marcela Santos, Gloria Balseca

**Affiliations:** 1 Department of Human Development and Family Studies, Utah State University, Logan, UT 84322, USA; kristin.murphy@usu.edu; 2 Institute for Disability Research, Policy and Practice, Utah State University, Logan, UT 84322, USA; eduardo.ortiz@usu.edu; 3 Facultad de Postgrado, Universidad Casa Grande, Guayaquil 090613, Ecuador

**Keywords:** child development in LMICs, nutrition, Ecuador

## Abstract

Many young children in Ecuador suffer from high rates of malnutrition and stunting that affect their long-term growth and development. Little is known about the dietary patterns of children from the Amazon region who experience some of the highest rates of stunting (height-for-age) within Ecuador. Semi-structured interviews were conducted with 50 mothers of young children living in the Ecuadorian Amazon. In addition to descriptions of overall dietary patterns, three themes emerged from the interviews relating to strengths mothers have in feeding their children healthy diets: knowledge, autonomous and independent children, and supportive and responsive parenting. Five themes were found relating to barriers mothers have in feeding their children healthy diets. The first four themes concerned barriers (lack of knowledge of healthy foods, lack of access to healthy foods, not enough money, and child’s health) related to multidimensional poverty. All these influenced the last theme found, namely, how difficult of an eater the child was. The implications of intervention efforts to reduce undernutrition and promote children’s development by building on specific family and community strengths and identified barriers are also discussed in this paper.

## 1. Introduction

Children in Ecuador suffer from high rates of undernourishment and stunted growth (low height-for-age) [1]. In fact, Ecuador has one of the highest rates of undernourishment in Latin America with 4.8 million, while 24% of children under the age of five are stunted in growth [2]. More specifically, differences in growth between the poorest and richest children in Ecuador are apparent. Of the richest 20%, 14% of children under the age of five are stunted and that number jumps to 37% amongst the poorest 20% of children [2]. When children experience chronic or reoccurring undernourishment early in life, their bodies do not have enough nutrients to grow to their full potential, resulting in stunting [3]. As such, stunting is often used as an indicator of undernourishment in the literature, and these poor health indicators have been shown to have negative implications on children’s development, individual productivity, and community and national productivity in adulthood in a variety of Latin American countries and on a worldwide scale [4,5,6,7,8].

Beyond the high rates of undernourishment and stunting that have been observed in the past in Ecuador, the country is currently undergoing a transition to a more urbanized society, and this transition puts Indigenous and rural-living people at an even higher risk of undernourishment and poverty [1,9,10]. This relationship between urbanization and malnourishment was predicted over twenty years ago by Barry Popkin [11], who found increases in overweight and obesity at the beginning of world urbanization across low- and middle-income countries (LMICs). When children experience malnourishment early in life, their bodies learn to conserve fat and energy more than those who do not experience malnourishment early in life [12,13]. Since more westernized diets are characterized by higher levels of processed carbohydrates and fat, children who experience malnourishment and then switch to a more westernized diet are at a higher risk of having growth problems like overweight and stunting [12,14]. Part of this urbanization process that is influencing Ecuadorian children’s growth and development is market integration. Traditionally, Indigenous groups have been self-sustaining and have not participated in the market. Some examples of market integration include selling agricultural products or animals and wage labor outside of the local community. Houck and colleagues [4] found that amongst different communities of the same Indigenous group, those who live farther away from main roads had less market integration and higher rates of stunting and malnourishment. However, when examining differences between Indigenous groups, they found that for some groups, traditional diets and less market integration was associated with lower rates of stunting and malnourishment. Ecuador has three national languages, many additional Indigenous spoken languages, and 14 different nationalities. There are also 24 different provinces that fit into four ecological regions: the mountains or highlands, the coast, the Amazon jungle, and the Galapagos Islands. The complicated and inconsistent relationships among market integration, diet, and child development that previous researchers have described are likely due to the cultural and ecological diversity within the country. What works for one group of people in one part of Ecuador may not work for another group or in another part of the country.

Over one million people of Ecuador’s seventeen million population are Indigenous and almost one quarter of those Indigenous populations live in the rural Amazon area [15]. The urbanization of these populations may be impacting their diets, as their access to foods is likely changing. The current study uses a qualitative approach to explore the nutritional patterns of families with young children from the rural Amazon region in Ecuador. Specifically, the purposes of this study were to examine the children’s dietary patterns, identify the strengths the families currently utilize from the varying levels of their environment, and describe the barriers they face to providing healthy diets to their children. Children who are stunted have decreased human capital and are less likely to develop to their full potential [16]. Understanding what children from the Ecuadorian Amazon eat and the influences on their dietary patterns will help us understand what may be impacting their physical growth. This information can be used to inform future efforts to develop effective and useful interventions to help families in the Amazon prevent stunting and promote optimal development in their young children.

### 1.1. Dietary Diversity

Researchers have shown that the first 1000 days of life, from the time of conception to the child’s 2nd birthday, is a critical period for the children’s development [2,17]. Although it is possible to make up some developmental losses that originated in a child’s early life [18], it is best to avoid those losses whenever possible in the first 1000 days of life. Some researchers disagree on what it is about nutrition and diet specifically that influences stunting and development in young children. First, researchers have found that micronutrient consumption, such as B12, iron, iodine, folate, and calcium, during prenatal development (via the mother’s consumption) and the first few years of life act as a protective factor for children’s healthy physical growth and cognitive development in low- and middle-income countries all around the world [19,20]. Fewer studies examine macronutrient intake but several researchers report that it is actually macronutrient consumption which most commonly comes from animal-sourced foods (e.g., milk, meat, eggs, and fish) that is a protective factor against stunting and developmental delay [21,22]. A meta-analysis suggests that single micronutrient interventions have no or very small effect on children’s physical growth [23]. Furthermore, researchers working in Ecuador have found that it is a combination of micro- and macronutrients that act as a protective factor [24], and it may be that children need a certain amount of diet diversity to receive this combination of micro- and macronutrients and protect themselves against stunting [25,26].

Dietary diversity refers to the number of food groups an individual consumes in a day. Eating a variety of foods from a variety of food groups increases the amount and variety of nutrients a child receives and protects against stunting. Stunting during early childhood has long-term negative impacts on children’s development resulting in increased morbidity and mortality, lower levels of neurodevelopment and physical development, and a loss of developmental potential [27,28]. According to UNICEF [2], two in five children aged 6 to 23 months in Latin America do not eat the suggested minimum dietary diversity, 30% are nutrient-deficient in the essential nutrients typically consumed through animal-sourced foods, and 20% do not eat any fruits or vegetables. Furthermore, Ecuador has the second highest rate of childhood stunting, wasting, and overweight in Latin America, trailing only Guatemala [2]. Increasing the amount of diet diversity young children receive in this country may decrease the high rate of stunting they are experiencing. However, given the great ecological and cultural diversity in Ecuador, it is also important to understand the macrosystem and the underlying and basic causes of undernutrition by region before trying to create and implement an intervention aimed at helping reduce stunting.

### 1.2. Amazon Region of Ecuador

The Amazon region includes eight different countries in South America. Ecuador contains only about 2% of the entire Amazon basin; however, this 2% makes up almost 35% of the total land and 80% of the forested land in Ecuador. This region covers the most eastern inland section of the country. Participants for this study are from one of the six Ecuadorian Amazon provinces, Sucumbíos. Sucumbíos is in the far northeast corner of Ecuador and has a total population of 176,000 people, the majority of whom live in rural communities. Specifically, participants live in a rural area best known for its National Biological Reserve and oil field. A large portion of those who live in this area belong to the Indigenous Kichwa group. Since the early 1990s, the Kichwa people have fought for their rights, land, and way of living within the country; however, over time the government has granted foreign companies access to the Amazon. Most notably, the amount of oil drilling and extraction has continued to grow in this region of Ecuador. Urbanization impacted families when oil was discovered in the region, oil companies began to build roads to access the oil, and then migrants began settling there [29]. The heads of households in this region have only six years of formal education on average [30]. The low levels of education in the Amazon make it difficult for individuals there to overcome the barriers of market integration they face even though higher market integration has been shown to result in higher wages [31]. 

### 1.3. Family Practices

Although the nutritional intake of young children in Ecuador directly influences their physical growth and development, integrated efforts targeting multiple aspects of children’s environments are more effective and sustainable and can help children reach their full developmental potential [32,33,34,35]. In the State of the World’s Children 2019 publication, UNICEF stated that “supportive systems” need to be utilized and coordinated with nutritional intervention approaches to help children reach their full developmental potential [2]. One specific aspect of children’s environments that impacts their growth and development is the cultural beliefs and familial practices surrounding food. These beliefs and practices can include important cultural foods that families feed their children, how parents prepare and cook food for their children, and the routines families have surrounding food and mealtimes [36,37]. Indeed, traditional foods and practices in Latin America, including lower levels of market integration, can be beneficial for children’s development because they are often nutrient-dense and unprocessed, such as fruits, vegetables, and animal-sourced foods like meat and eggs [38]. However, traditional foods are not always nutritious or unprocessed. Of particular concern in Latin America is a fermented drink called Chicha. Chicha is a traditional drink consumed in many rural parts of Latin America that may create a risk for children’s development due to its alcohol content [39].

Some researchers have found that market integration has resulted in Ecuadorian families relying more on low-nutrient-density, high-caloric foods that do not provide children with enough dietary diversity but are commonly found in stores [1,4]. However, it is unknown how Ecuador’s urbanization is impacting the families living in the Amazon region because families often do not have easy access to stores and markets. It may be that parents do not have enough knowledge about healthy diets and how to provide healthy diets, or enough consistent income to provide healthy diets for their children due to the general low levels of education that are common amongst families in the Amazon [40,41]. Ramirez-Luzuriaga and colleagues [42] found that children from low-income, low-education, or Indigenous groups in Ecuador had disproportionately high rates of malnourishment compared to middle- and high-income and education and majority ethnic groups. In addition to urbanization, this lack of knowledge and income may be contributing to their dietary choices, impacting their children’s development.

Also contributing to their children’s development is the parents’ knowledge, or lack thereof, about the importance of having a routine. This lack of knowledge and consistent access to healthy food choices may also interfere with daily routines surrounding their consumption (e.g., regular family mealtimes). Indeed, families may eat food whenever they have it, because they may be food-insecure and not have consistent access to food or the income to consistently purchase food [43]. This may also be contributing to the high rates of stunting in the country, because children are fed whatever food they have access to rather than a variety of nutrient-dense foods, or they simply go without food for parts of the day. Parents may be more concerned about being able to feed their children at all and providing them with enough calories rather than being able to consider the variety and amount of nutrients. For example, one common cultural practice in Ecuador is to breastfeed children [2]. Researchers have shown that breastfeeding has positive health and developmental benefits for children. The World Health Organization (WHO) recommends that infants worldwide be exclusively breastfed for the first six months of their lives 3]. Infants who are breastfed have a lower risk of being overweight or obese, or having respiratory infections, sudden infant death syndrome, and cognitive delays [44,45,46]. However, mothers from rural Ecuador report choosing to breastfeed their children primarily because it is cheap and convenient, not because it provides all the nutrients children need for healthy development nor because it is a protective factor against any health and developmental concerns. This is concerning for multiple reasons. First, mothers are breastfeeding out of convenience, or not exclusively breastfeeding for six months, and they are not aware of or following the WHO guidelines for exclusive breastfeeding, which can have negative health impacts on their children. Second, when mothers do introduce complementary foods to breastmilk, they are not choosing the appropriate nutrient-dense foods that infants need to grow and develop [47]. These mothers might be malnourished or stunted themselves, and so they cannot pass much needed nutrients on to their children through breastmilk, resulting in an intergenerational transfer of malnourishment and stunting [48,49]. Understanding in more depth these nutritional practices, the strengths that parents already have, and the barriers that parents face can help inform future interventions targeted at increasing the parents’ knowledge and access to a diverse and nutritious diet for their children. This subsequent increase in their children’s dietary diversity could reduce stunting and help their children reach their developmental potential.

## 2. Materials and Methods

### 2.1. Qualitative Approach and Research Team Characteristics

This study was performed from a constructivist point of view as we aimed to construct the dietary patterns, strengths, and barriers related to children’s healthy diet from the mothers’ viewpoint. An inductive approach was used to gain an understanding of the dietary patterns. This was followed by a deductive approach informed by a conceptual framework of the determinants of children’s undernutrition described in the following section. The fourth and fifth authors along with all four research assistants are Ecuadorian and have lived in Ecuador for their entire lives. The third author is Ecuadorian, grew up in Ecuador, and graduated from law school before moving to the United States. The second author has worked with her Ecuadorian colleagues for more than a decade and frequently travels to Ecuador to collaborate on research projects. The first author worked under the direction of the remaining authors as she designed the study and completed her doctoral program. The fourth author along with the four research assistants coded the interviews and actively participated in interpreting the findings. The fifth author has worked with community programs in the two townships where recruitment took place.

### 2.2. Theoretical Framework

Bronfenbrenner’s bioecological theory, specifically the Process–Person–Context–Time (PPCT) model, posits that the interaction between processes and individual factors drives development in a variety of contexts and across time [50]. This study focuses on the context under which an individual experiences these proximal processes surrounding food and nutrition. Bronfenbrenner describes the context in exceedingly distant systems that make up an individual’s environment. The microsystem includes those that make up an individual’s direct environment. The mesosystem describes how different microsystems interact. Factors that are not in the individuals’ immediate environment but are still an influence, such as parents’ work, make up the exosystem. The macrosystem includes broader cultural and societal influences, and the chronosystem considers the historical time that influences how an individual develops. This framework brings attention to a variety of different factors, from the child’s characteristics such as gender and level of malnutrition, to the home environment, and familial and cultural practices surrounding nutrition and development.

### 2.3. Conceptual Framework

The United Nations Children’s Fund (UNICEF) developed a conceptual framework of child undernutrition to explain the broad and multifaceted causes, consequences, and impacts of childhood undernutrition [51]. This model includes immediate, underlying, and basic causes of malnutrition that correspond with Bronfenbrenner’s contextual systems. In developing this model, UNICEF aimed to include all the broad, multifactorial causes as well as short- and long-term impacts of child undernutrition that apply to children from all around the world.

This study used UNICEF’s conceptual model as a guide to describe and understand the potential factors that may be influencing children’s nutritional intake, growth status, and cognitive development in the Ecuadorian Amazon region. Since we did not know how this model functions with families from this area, more detailed qualitative data will help to guide future research and intervention efforts, helping focus these efforts on the parts of the framework that may be more strongly impacting this specific population. Indeed, there are many different influences and factors that interact to result in the diet an individual child receives, and these can vary greatly depending on the environment. For example, a child repeatedly interacts with food daily and if their diet does not consist of healthy, nutrient-dense foods (i.e., immediate cause of undernutrition), the child will be at risk of growth and developmental delays. However, young children do not often choose what they are going to eat; rather, their parents select what foods to grow, purchase, and offer in the home environment based on their access to food (i.e., microsystem, underlying causes), and those decisions are often influenced by the parents’ education and income levels (i.e., exosystem, basic causes), where they live, and cultural caregiving practices and beliefs (i.e., macrosystem, basic causes). Although this UNICEF model includes many factors that have the potential to influence these children’s development, it is beyond the scope of this study to examine them all. Instead, this study focuses on describing the nutrition and caregiving practices surrounding food that impact Ecuadorian children’s rates of stunting (i.e., portions of the immediate, underlying, and basic causes).

### 2.4. Participants

Fifty mothers with young children attending their community center-based program, Centro de Desarrollo Infantil (CDI), in two townships were recruited to participate in this study. The sample was a convenience sample with recruitment continuing until a sample size of 50 (25 in each township) was reached. Based on similar qualitative research conducted in Ecuador, a sample of fifty was chosen because we believe it provides enough data for saturation to be reached [52,53]. Participants’ demographic information, including parent education levels, household income, and child age was measured using UNICEF’s Multiple Indicator Cluster Surveys (MICS). The highest level of education mothers had completed was almost evenly split between primary (44%) and secondary (54%) schooling, with one mother having completed more than secondary school (2%). Household income ranged from zero to USD 700 per month, with an average of USD 178.20 per month. Mothers were 26 years old on average. The youngest mother was 17 years old and the oldest was 50. Six mothers were adolescents, and twenty-four were under the age of 26. Children were between the ages of 11 and 38 months old, with a mean of 26 months. A total of 58% of children were female. The majority (88%) of households spoke both Spanish and the local native language, Kichwa, and 88% of families identified as belonging to the Kichwa ethnic group. The remaining 12% identified as ‘mixed’ ethnicity.

While the sample can be broadly defined as a convenience sample, we aimed to include participants with differing family structures, income levels, education, and socio-economic status. Children of adolescent mothers were also prioritized, as Ecuador has one of the highest rates of adolescent pregnancy in Latin America and these children are particularly vulnerable to experiencing developmental delays due to the increased risk factors they experience [54,55]. The sample demographics were representative of the programs where they were recruited and reflect the high rate of adolescent mothers in the country. Participants were only excluded if their children have severe disabilities that influences their eating habits, physical growth, or cognitive development. 

### 2.5. Measures

Each participant was asked about their children’s dietary patterns and what influences the nutritional choices they make for their children during semi-structured interviews. UNICEF’s framework of undernutrition was used to guide the creation of the initial semi-structured interview questions. The questions were then translated into Spanish and remained in Spanish while they were reviewed and revised by several members of the research team, including two members in Ecuador and the author who is originally from Ecuador. The framework, theory, and previous literature, and the collaboration and input from the various members of the research team led to the development of the final interview questions. These general questions addressed the length of time and reasons for breastfeeding, a child’s typical day including meals and specific foods eaten, the parent’s knowledge of a healthy diet, and any difficulties feeding children healthy foods. The questions were developed in Spanish and included terms used by the families. For example, the word “meals” is an English word that has a vague definition, but the Spanish word ‘comidas’ was used during the interviews because it is culturally relevant to this group of families, as snacking is reportedly not common, and it is a clear and well-understood word by participants.

Mothers were also asked about the children’s consumption of specific foods in the week prior to the interview. Specifically, these questions asked how often the child drank Chicha and ate fish, fruit, vegetables, and processed foods (e.g., potato chips, cookies, and pasta). Follow-up questions asked mothers why they fed their children each of the foods to learn more about what influences children’s dietary patterns for these particular foods and food groups. 

### 2.6. Procedures

Each interview was conducted in Spanish and was audio- and video-recorded. Participants chose to be interviewed in their homes or in the child development center that their children attend. One interviewer, who is familiar with the communities and trained in standardized interview procedures, conducted all interviews. Once reliable, the interviewer contacted mothers from the Amazon region through word of mouth and the child development centers where she works. Each mother was given an informed consent form, had the purpose and procedures of the research study explained to her, was asked if she had any questions, and was asked if she would like to participate in the study prior to starting the interview. The interviews were transcribed and checked by the research assistants who coded the interviews. Data analysis did not begin until all interviews were conducted and transcribed.

### 2.7. Data Analysis

This study has a descriptive design. NVIVO, a qualitative data analysis program, was used to perform the analysis of the data. This program helped organize and maintain the data with codes and notes for every member of the research team to view and edit. This program made creating summaries and visualizations quick and easy, and was used by the research team in Ecuador to code the interviews in their native language, Spanish. Each participant was assigned an ID number to preserve anonymity. The project was approved by an ethics board in Ecuador prior to conducting any interviews, and was approved by Utah State University’s Institutional Review Board (IRB) before analysis began.

First, data collected to better understand children’s dietary patterns were analyzed using an inductive thematic content analysis approach [56]. This approach allows researchers to create broad ideas based on their specific data and results [57], and to identify themes that we may not have initially considered for a phenomenon with limited research. To ensure the accuracy of coding, research assistants living in Ecuador working with the faculty author from the university in Ecuador were recruited to transcribe and code the interviews in Spanish. This helped ensure the details of the participants’ culture were not misrepresented or incorrectly understood. A team of two research assistants were trained in inductive thematic content analysis and ethical standards of research. Both research assistants reviewed and coded every interview. This open coding phase was completed in Spanish and continued until all interviews were coded and data saturation was reached (no new themes were identified in the final five interviews). Codes were then compared between coders to ensure accuracy and trustworthiness. Two of the authors along with the research assistants then engaged in axial and selective coding phases where codes were related to one another and grouped into broader themes and categories. These were then translated into English and a bilingual member of the research team who is from Ecuador verified the translations and that cultural beliefs and values were not being lost in translation. The results from these inductive thematic content analyses are presented in Section 3.1 of the Results. 

**Figure 1:**
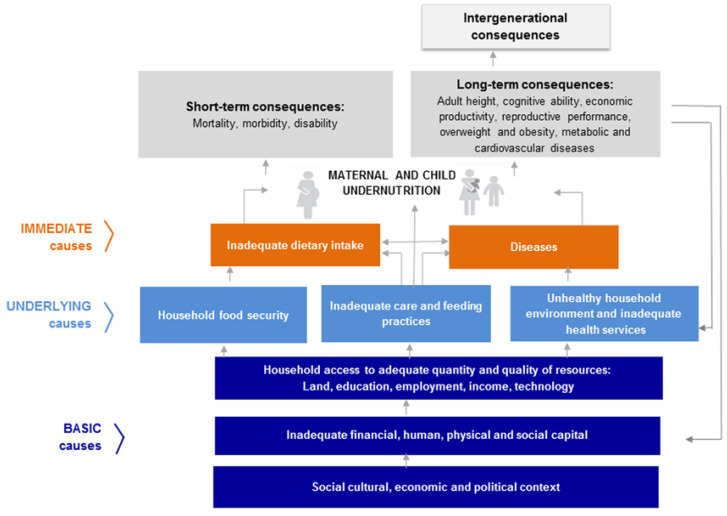
Conceptual framework of the determinants of child undernutrition used to guide this study [51].

Following the inductive content analyses, the UNICEF model presented in Figure 1 was used to guide deductive thematic content analyses. Specifically, the strengths and barriers to providing children with healthy diets were analyzed by a team of two additional research assistants using a deductive thematic content analysis approach [56]. This is used when researchers want to use existing theoretical ideas or models in a new context, such as the UNICEF model guiding this research. The research assistants were trained in this deductive method and asked to code the interviews by looking for two main categories, strengths and barriers. All interviews were coded and data saturation was reached (no new themes were identified in the final five interviews). Codes were then compared between coders to ensure accuracy and trustworthiness. Two of the authors along with the research assistants grouped the codes into broader themes within each category. Themes and categories were then translated into English and the bilingual member of the research team checked the translations for errors. The results from these deductive thematic analyses are presented in Section 3.2 and Section 3.3 of the Results.

## 3. Results

### 3.1. Dietary Patterns

Several themes emerged from the inductive thematic content analysis used to learn more about the adequacy of the typical dietary intake and why mothers fed children specific foods. First, mothers shared how breastfeeding is normal. Mothers exclusively breastfeed for at least six months and breastfeed for at least one full year. In only three cases did the mother report that she did not breastfeed for at least six months, and that was because she could not. In each of those cases, the mother reported that if she were physically able to breastfeed, she would have. While this is a good, health-promoting practice, mothers reported that they chose to breastfeed not for the positive benefits to the mother or child, but rather because it is a common cultural practice. Several mothers stated they breastfed their child because ‘it is natural’. Others reported that they chose to breastfeed simply because their child needed to be fed and it provided calories to make them full. Furthermore, many mothers explained that they knew it was healthy for their child to be breastfed because ‘they say it is healthy for the child.’ Referring to society in general, doctors or other public figures of authority, mothers described how they did not know why it was healthy for the child or how the child was benefitting from being breastfed, but that they heard it was healthy so they believed it was. When mothers were asked why they chose to stop breastfeeding, they reported one or more reasons: first, and most commonly, because the child did not want to breastfeed anymore; second, because the child got sick and they had to stop breastfeeding; and third, because the mother got sick, got an infection, or stopped producing milk. However, the mothers did not initially express these details. They talked about how their ‘milk wasn’t good anymore’ and that caused them to stop breastfeeding. It was not until probed by the interviewer to describe in more detail that most mothers gave such examples of why they stopped breastfeeding. There were some mothers who discussed the health benefits and were educated on why breastfeeding was beneficial; however, this was a small portion of participants.

After asking about breastfeeding, the interviewer asked mothers about their children’s daily routines and what they normally ate in a day. The first and most notable theme found in this section of the data is that these mothers did not know what a routine is. The interviewer needed to explain it prior to receiving their response. Mothers talked about how routines are not a thing, they did not have any routines, they did not know what a routine was, and they did not know why it mattered or why a routine might be important for their children or their families. Still, there are some common things that families from this region reported doing. First, families eat three meals a day when ‘times are good’ and fewer meals when they are not. Second, these meals were not necessarily at a set time, or even around the same time each day. Mothers fed their children ‘whenever they need’, meaning that mothers fed their children meals whenever they asked for food or said they were hungry. When there was not enough food to have a meal and their child asked for something to eat, mothers would give them Chicha, a traditional, cultural drink primarily made of yucca or cassava and often left to ferment before drinking. Mothers often reported using Chicha as a meal replacement when the child was hungry and they did not have food to feed them. Mothers also reported feeding their children ‘refrigerio’. This is a common word used in the region where the data were collected and it is similar to eating a snack. Mothers fed their children ‘refrigerios’ throughout the day, when the child asked for one. This lack of routine did not only pertain to diets; when asked about routines in general mothers reported that their child sleeps, wakes up, eats, naps, bathes, and goes back to sleep again. They did not have routines as the word is typically used; rather, the only common things they did throughout the day were necessities such as sleeping and eating. A small number of mothers also talked about daily activities including playing, learning, singing, and visiting extended family, and an even fewer number of mothers said their children participate in community activities such as playing sports and feeding chickens. Overall, the responses to these interview questions were minimal and mothers gave short, non-explanatory responses. They often referenced the strong cultural practices; for example, when asked why they engage in a particular dietary pattern many mothers responded by saying it was what they learned from their mother or grandmother. 

Details about the specific foods that children had eaten were obtained through parent responses to the five interview questions about specific foods of interest with this group.

First, mothers were asked about Chicha. Many mothers reported giving their child Chicha because it is something ‘everyone drinks’, it is ‘good and takes away hunger’, ‘it is natural’, ‘it is part of the culture’, and ‘it is a custom to teach [their] children’. Seven mothers said their child had never had Chicha. The children who did drink Chicha had a mean age of 8.6 months when they first had it and they typically drank it 4 days per week. Several mothers reported giving Chicha to their child so they could ‘learn to drink it’ even though they explained that it made their child ‘hurt’ or not feel well, the child cried when they feed it to him/her, and some even vomited. Next, mothers were asked about fish. Eight mothers reported that their child does not eat any fish, while those children who do, eat it about 3 days per week. The most common reasons for feeding their child fish were because the child likes it, and because it has nutrients to help the child grow. The third most common response was that when mothers run out of the food they have purchased from the store, they must find their own food. As one parent put it, “fish is the way of the native people”. Children are taught from a young age how to fish so they can catch their own food. The next two foods mothers were asked about were fruits and vegetables. Only one parent reported that her child does not eat any fruit, and that is because the child does not like it. In contrast, fifteen mothers reported that their children do not eat vegetables and the most common reason why was because their children do not like them. Those who do, eat fruit five days a week and vegetables four days a week on average. Similar patterns were found for feeding or not feeding children fruits and vegetables: the child likes or does not like to eat them, they are healthy and help the child grow, and mothers do not have enough money to buy them at the store so their children eat what is easy for the mothers to grow themselves. For example, several mothers talked about their child eating papaya because it is a ‘local fruit’ so when the child sees it outside, they ask for it, and it is free and easily accessible for the parent to get it and feed it to the child. Lastly, mothers were asked about processed foods. Fourteen mothers reported that their child does not eat any processed foods. Children who do, eat them about 3 days a week. The majority of mothers shared a general knowledge that processed foods are not healthy or less healthy, but they are cheap, easy, and their children enjoy them so they feed them to their children anyway. When discussing processed foods, a common pattern emerged where the children eat more fresh food at the beginning of the month, or right after the mothers go to the market, and more processed foods towards the end of the month when the fresh foods have been eaten or gone bad.

This initial inductive content analysis helped us determine that the UNICEF model could be useful to increase our understanding of the factors that may be influencing children’s nutritional intake with this understudied population. The following sections summarize the results from the deductive content analysis that was used to identify existing strengths and barriers to feeding children healthy diets. The strengths and barriers coded inform the underlying causes and basic causes presented in the UNICEF model. 

### 3.2. Parental Practices Related to Children’s Healthy Diets

Mothers discussed several topics that pertained to strengths they already have (Table 1). Three main themes were found: knowledge, autonomous and independent children, and supportive and responsive parenting. Mothers had knowledge that eating healthy food is important to help their children grow and develop, that fruits and vegetables are healthy, and that sweets, fats, and processed foods are unhealthy. In addition, mothers talked about believing that it is the mother’s right to take care of her child to ensure the child develops in a healthy way. The last aspect of knowledge mothers discussed was that they perceived eating healthy food not to be difficult, including feeding their children a healthy diet. 

The second major theme that arose was that children are independent enough to do their own activities such as eat, get dressed, and get up on their own. Many mothers described this relationship as ‘apego’ which refers to the positive relationship between mother and child and means that the child is independent enough to not fully depend on the mother. The mother is able to eat a meal herself or accomplish other tasks when the child is physically able and willing to eat on their own. These positive child characteristics may be related to the last major theme found: supportive and responsive parenting. Mothers reported having positive interactions with their children on a daily basis, including picking them up, holding them, and hugging them. Additional positive interactions included ‘practica de aprendizaje’ or helping children learn or do school work, singing to their children, reading to them, and ‘interacciones’. The term ‘interacciones’ directly translates to ‘interactions’; however, this group of Ecuadorian mothers used it to specifically refer both to being physically close and having warm verbal communication with their children. This theme also encompassed more than just interactions between the mother and child. Mothers reported how the entire family, and sometimes the entire community, was involved in positive interactions with their children. Older siblings, cousins, and other extended family members often play with children, model behaviors, and help teach the younger children. Mothers reported this as something that does not just happen sometimes or for some families. They used the phrase ‘entre primos’ which directly translates to ‘between cousins’. 

### 3.3. Barriers Mothers Face in Feeding Their Children Healthy Diets

**Figure 2:**
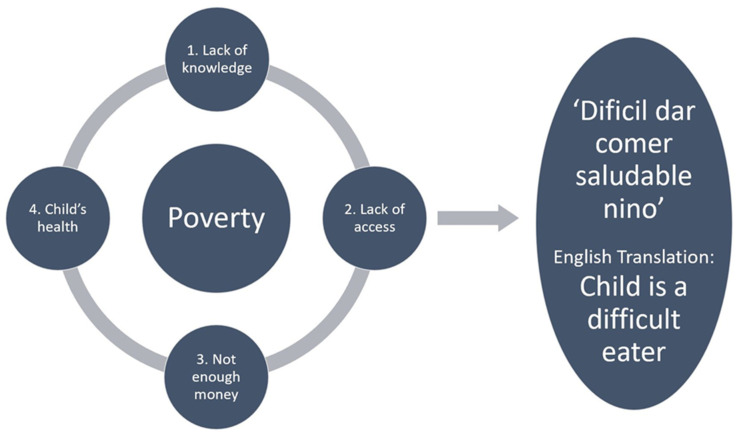
Barriers mothers face in feeding their children a healthy diet.

Despite having several strengths, mothers also faced barriers in feeding their children healthy diets and helping them grow and develop to their full potential (Table 2). Five different barriers were found amongst this sample of mothers, four of which fit under the broader theme of multidimensional poverty: lack of knowledge, lack of access, not enough money, and children’s health. The last barrier, the children being difficult eaters, appeared to be a result of multidimensional poverty which is the underlying mechanism through which the first four barriers may contribute to a child being perceived as a difficult eater. Each barrier and the relationship between them are displayed in Figure 2.

The first barrier mothers discussed was that they had a lack of knowledge regarding healthy diets and development. When asked what they know about healthy diets, several mothers reported that they did not know what a healthy or unhealthy diet is for mothers and for children. Second, mothers lacked access to healthy foods and a variety of foods. These families discussed their ‘escasez alimento’ or their ‘food shortage.’ They reported eating the foods that are locally available because they do not have the funds or ability to frequently travel to a grocery store or market that has a wider variety of foods. This leads to the third barrier mothers face, not having enough money to eat a healthy diet or provide their children with a healthy diet. In their interviews, even mothers who had the knowledge of what a healthy diet is discussed that they did not have enough money to provide that type of diet to their children. Many families said ‘compra primeros dias’, meaning that they go to the market once a month, they purchase what they have money for, which is never enough to purchase everything they want, and when the food is close to running out towards the end of the month, they must eat local foods or the less healthy, canned foods they purchased at the market at the beginning of the month. The fourth barrier mothers face is their children’s health and illness. ‘Problemas salud niño’, as many mothers put it, referred to their children getting sick and that making it difficult to feed them a diverse and healthy diet. When their child had diarrhea or was sick, they had to feed their child a plain, simple diet that they are used to so as to not upset the child’s digestive system any more than it already was. Several mothers explained that feeding their children a lot of vegetables or fresh foods that they are not used to eating would make them sick.

These four barriers all relate to and fit underneath the broad umbrella theme of multidimensional poverty that extends beyond a lack of money (e.g., nutrition, education, and clean water/sanitation) [58]. The level of poverty these families live in contributes to their lack of knowledge, lack of access, not having enough money, child’s health problems, and contributes to the practices families engage in surrounding these four barriers. Furthermore, it is this multidimensional poverty and subsequent barriers mothers face that seems to lead to the child becoming a difficult eater. This was exemplified by many mothers who said ‘difícil dar comer saludable niño’ which means it is difficult to feed their children healthy diets. Mothers discussed how their ‘niño come lo mismo’ or their child likes to eat the same thing all the time. When discussing and examining these barriers during the axial and selective coding phases, research assistants made a point to state that the child becoming a difficult eater was not necessarily a responsibility of the parent for not teaching the child or of the child due to their temperament. It may be due to a combination of the two, but more importantly, the research assistants reported that it seemed to be caused by the multidimensional poverty these families experience.

## 4. Discussion

### 4.1. Summary of Dietary Patterns

Given Ecuador’s high rate of undernourishment [2], this study used UNICEF’s conceptual model as a guide to describe and understand the potential factors that may be influencing children’s nutritional intake in the Ecuadorian Amazon. Mothers of young children were interviewed to gain information regarding the dietary patterns of their young children. Several dietary patterns were found in relation to breastfeeding, what children do on a typical day, and what children typically eat. However, the responses to the interview questions were often short and non-explanatory with many mothers saying that the behavior or dietary pattern was what they learned from their mother or grandmother. This finding supports previous research about the strength of cultural practices that are passed down through the generations, and that families engage in dietary practices because it is what they have learned from older relatives, and not necessarily because it is the best or healthiest for their child [2]. Almost all mothers reported breastfeeding their child and many stated that it was healthy but they did not know why it was healthy for the child other than what their mother or grandmother told them. Similarly, when mothers were asked how many times their child typically eats in a day, the research team expected mothers to report the number of meals each child eats. Instead, mothers reported that they eat “whenever we need” and “whenever we’re hungry.” This lack of routine and pattern may reflect inconsistent access to food [45] and a lack of knowledge of the dietary needs of children for optimal growth. 

Information regarding dietary patterns was gleaned from the interviews in relation to the five specific foods mothers were asked about. Fewer children in this sample typically eat any fruits or vegetables (70%) compared to the 80% in Latin America who eat any fruits or vegetables [2]. However, in this sample 84% of children eat fish, which is a type of animal-sourced food, but in the sample from Latin America only 70% eat animal-sourced foods on a regular basis. It is a combination of these and other foods that provide children with both micro- and macronutrients that act as a protective factor against stunting [26,27]. More research is needed, but it seems that children in this area of Ecuador are receiving animal-sourced foods, which provide them with necessary macronutrients, but they may not be eating enough fruits or vegetables, which provide their bodies with micronutrients necessary for growth and development. 

Lastly, mothers were asked about two additional foods: Chicha and processed foods. The vast majority, 86% of this sample of young children, drink this traditional, fermented drink. Many mothers discussed believing it was healthy, feeding it to their child because it was filling, or because it was part of the culture. Mothers began feeding this to their child when they were as young as four months old even when they did not want it or like it. These responses represent the strong cultural practices amongst this group of families. Even though their children may not like or want to drink Chicha, they are still expected to ‘learn to drink it’ because it is part of the culture. This is concerning, as Ramirez-Ubillus and colleagues [39] found that the alcohol content of this drink is a risk factor for children’s development. Future research should examine the alcohol content of the Chicha fed to children and examine the specific negative and positive impacts of this drink. With this additional knowledge, future interventions could provide information to mothers on how it might be impacting their children, as well as other ways mothers could prepare and serve Chicha with lower or no alcohol content. When asked about processed foods, 28% of mothers reported that their child did not eat processed foods, while those who do, eat them about 3 times a week. The reasoning mothers gave for eating or not eating these foods related back to access to fresh foods and household income. Families’ access to fresh foods is part of UNICEF’s underlying causes, while household income is considered a basic cause [17,18]. This demonstrates how multifaceted and complex the barriers are that these families face. It is not just one issue or even multiple issues that relate to one system, that lead to the food that children eat on a daily basis. Rather, there are many different issues at a variety of levels and in different parts of their lives that come together to influence parents’ decisions surrounding food and children’s food intake [17].

### 4.2. Strengths and Barriers in Relation to Potential Intervention Efforts

An understanding of the basic and underlying causes that contribute to children’s healthy diets or undernutrition could help focus intervention efforts in communities with limited resources. Mothers discussed several strengths they already have when it comes to feeding their children healthy diets and encouraging strong growth and development. Themes included knowledge, autonomous and independent children, and supportive and responsive parenting. These three strengths all represent areas future research and intervention efforts could focus on to help improve children’s nutrition and subsequent development. Similar to the dietary patterns found, the information mothers provided on each of these three themes was quite general. For example, mothers knew that feeding their child a healthy diet is necessary for them to grow and develop, but they did not know why or how eating healthy caused their child to grow and develop. This emerged as both a strength and a barrier for this group of mothers. The knowledge that mothers have, or lack thereof, is an example of a basic cause in UNICEF’s framework of undernutrition [18]. Educating mothers on this topic may not only help them become more committed to feeding their child healthy foods but also encourage them to problem solve and think more critically about feeding their child a diverse and well-rounded healthy diet. 

For children of this age range, having a healthy level of autonomy and independence was important to mothers. As indicated by the mothers in this study, this may be impacting children’s diets because children who are not able and willing to eat on their own may have to wait until the mother has time and can feed them. Mothers also discussed engaging in supportive and responsive interactions with their children. Observing and learning more specifics about the behaviors mothers engage in could guide intervention efforts to encourage mothers to focus on and engage in such behaviors more often. These interactions represent an underlying cause according to the framework of undernutrition [18]. Although they are not directly related to dietary intake, mothers recognize the importance of these positive interactions, and this supports previous research indicating that supportive systems need to be included in nutritional interventions to help children reach their full developmental potential [2,37]. Furthermore, the common practice of older siblings and cousins modeling behaviors for younger children could be used to give knowledge to mothers about the entire context that a child develops under. This supports Britto and colleagues’ [32] findings that nutrition interventions that include other aspects of the children’s environment are the most effective. To execute such an intervention with this group of families requires careful consideration to be culturally sensitive and build on community and family strengths.

The additional barriers of lack of access to food, not enough money, and child health all relate to multidimensional poverty and, as explained by the mothers, result in more difficulty in feeding the child a healthy diet. Due to the multidimensional poverty these families experience, they eat fewer healthy foods and more foods they have traditionally grown or caught. Many of the parents go to the market once a month to buy food. Whereas this market integration has helped in some ways, it may have also negatively impacted these children’s diets as parents purchase processed, canned, and less healthy foods from the market because they are cheaper than fresh foods and they last longer than fresh foods. Market integration has allowed parents the opportunity to go to the store and purchase a wider variety of foods than their ancestors ate from farming and fishing; however, most of these families do not have the means of transportation or money to go to the market more than once a month. Just as Houck and colleagues [4] found mixed results on the impact of market integration, this group of families experience both some benefits and some drawbacks. If families were able to increase their income and have better transportation to and from the market, they might be able to take advantage of the wider variety of fresh foods available and provide a more diverse and nutrient-dense diet to their children. As it is now, children’s bodies are likely learning to conserve fat and energy more than needed, as families sometimes do not have enough food to feed the child a healthy diet [12,13]. Similarly, the finding from this study indicating that children get sick or develop diarrhea when eating new, fresh foods such as vegetables, may be due to the lack of exposure over the first few years of their life, resulting in them becoming pickier and more difficult eaters because their bodies have adapted to and learned how to conserve the energy and nutrients they are used to eating [12,13]. As such, helping these families move out of poverty could help their children become less difficult eaters. This exemplifies how changing an underlying basic cause in UNICEF’s conceptual framework could have a large impact on the direct causes of undernutrition, greatly impacting children’s development.

### 4.3. Study Limitations

The results seek to highlight the dietary patterns, strengths and barriers mothers in the Ecuadorian Amazon experience as guided by the UNICEF conceptual framework of the determinants of child undernutrition. The convenience sampling from two townships in the Amazon region of Ecuador limits the generalizability of the findings. Another limitation is the reliance solely on mothers’ reports of dietary patterns. While providing important information about their experiences, including interviews with fathers and other caregivers, observing children eating throughout a typical day, and measuring the nutritional content and amount of the food eaten would provide data triangulation and strengthen this study. 

### 4.4. Implications and Future Directions

Future research and intervention efforts should focus on nutrition education and strategies to improve the economic resources that would enable these families to provide healthier diets for their children. However, the strong cultural beliefs and practices will need to be considered as intervention efforts must be individualized to the unique wants and needs of members in the community [59]. The results from this study identify current cultural practices and family strengths that could inform intervention efforts to improve children’s diet diversity and promote optimal development. First, encourage families to begin to cultivate other foods that can be easily grown in the area, educate parents on how they can grow and prepare them, and strengthen the green and self-sufficient economy they already engage in. Second, give information about what routines are and why they are important, using concepts and examples from the culture. Third, strengthen parenting practices by validating the positive parent–child interactions, and then encourage parents to engage in frequent positive interactions as they complete their daily tasks. Finally, educate parents on problem-solving skills and ways of thinking as well as more detailed information about why nutrition matters, what a healthy diet looks like, and specifically how it influences their child’s growth and development. These and other intervention efforts guided by UNICEF’s conceptual framework that build on family and community strengths could help address the high rate of undernourishment in Ecuador and improve children’s health, development, and individual productivity in adulthood [5,6,7,8].

## Figures and Tables

**Table 1:** Strengths in promoting healthy diets, along with example quotes by the mothers given in Spanish and translated into English.

Original Quote in Spanish	English Translation
Parent Knowledge	
Bueno, tengo entendimiento que son frutas, verduras, carnes, pescado, pollo y purés. Las mamás, por ejemplo, más frutas deben comer, leches, yogures, quesos. …eso es el derecho de la madre para cuidar a un hijo para que crezca sanamente. No, no es difícil. Después que hago, sí se los come.	Well, I understand that they are fruits, vegetables, meat, fish, chicken, and purees. Moms, for example, should eat more fruits, milk, yogurt, cheese. …that’s is the mother’s right to take care of a child so s/he can grow healthy. No, it is not difficult. After I do (that), s/he does eat them.
Autonomous and Independent Children	
…ella come sola, no le gusta que le ayude. Juega, con su ñaña juega de vez en cuando.	…she eats by herself, she doesn’t like that I help her. S/he plays with her sister from time to time.
Supportive and Responsive Parenting	
Sí, pasamos ahí en la cama, le gusta jugar ahí saltando. Juego, la paseo. Cuando él está así con ganas de jugar coge la pelota, jugamos y nos divertimos entre los dos, entre todos mejor.	Yes, we spent time there on the bed, he likes to play jumping there (on the bed). I play, I walk (with him/her). When he is like that, wanting to play, he picks up the ball, we both play and have fun, (and it is) better all together.

**Table 2:** Barriers mothers face in providing healthy diets, along with example quotes by the mothers given in Spanish and translated into English.

Original Quote in Spanish	English Translation
Lack of Knowledge of Healthy Foods	
Mothers’ response when asked what they know about a healthy or unhealthy diet: No sé, la verdad no.	I don’t know, not really.
Lack of Access to Healthy Foods	
Bueno, cuando uno hace compras los primeros días, se tiene todo, pero ya después, al fin de mes, ahí no hay cómo porque se van acabando las cosas.	Well, when you shop (groceries) the first few days (of the month), you have everything, but later, at the end of the month, there’s no way because things are running out.
Not Having Enough Money to Eat a Healthy Diet	
Después de que tenga dinero sería para mí fácil.	After I have the money, it would be easy for me.
Children’s Health and Illness	
Ciertos vegetales les afloja el estómago. Es que no le hace bien en el estómago. …lo que él no le gusta, así, leche, le hace daño pero igual necesita la leche.	Some vegetables loosen their stomach (diarrhea). That is not good for his/her stomach. … what he doesn’t like, for example, milk, it hurts him but he still needs milk.
Multidimensional Poverty	
Cuando se hace lo mismo y lo mismo como que se aburren. El mismo sabor. Sí, el mismo sabor. Combinarlos con otras cosas. Sí, porque los niños botan, no les gusta.	When you do the same thing and the same thing, you kind of get bored. The same flavor. Yes, the same flavor. Combine them with other things. Yes, because the children throw away, they don’t like it.

## Data Availability

The data presented in this study are available on request from the corresponding author. The data are not publicly available due to ethical approval guidelines.
